# Dogs’ Behavioural Responses to Dog-Assisted Interventions: A Field Study

**DOI:** 10.3390/ani16071063

**Published:** 2026-03-31

**Authors:** Sandra C. Haven-Pross, Anna L. Jens, Kyra N. Maarleveld, Peter van Honk, Manon de Kort, E. Kathalijne Visser

**Affiliations:** 1 Department of Applied Research, Aeres University of Applied Sciences Dronten, De Drieslag 4, 8251 JZ Dronten, The Netherlands; a.jens@aeres.nl (A.L.J.); k.maarleveld@aeres.nl (K.N.M.); p.van.honk@aeres.nl (P.v.H.); k.visser@aeres.nl (E.K.V.); 2 Behavioural Science Institute, Radboud University, P.O. Box 9104, 6500 HE Nijmegen, The Netherlands; 3 Department of Applied Biology, HAS Green Academy, University of Applied Sciences, Onderwijsboulevard 221, 5223 DE ’s-Hertogenbosch, The Netherlands; m.dkort@has.nl

**Keywords:** animal-assisted services, animal welfare, dog–human interaction, dog affective states

## Abstract

Animal-assisted services (AASs) are increasingly integrated into healthcare, education, and social support settings. However, empirical evidence on the emotional well-being of participating dogs remains limited. This study investigates how dog, session, handler, and client factors influence dogs’ affective states during animal-assisted activities (AAAs), education (AAE), coaching (AAC), and therapy (AAT). A total of 837 sessions involving 63 dogs and 30 handlers were observed, with behavioural scoring and statistical analyses used to analyse the data. Principal Component Analysis then identified key affective components, including playfulness, comfort, anxiety, and uncertainty, which explained 45–61% of the variance. Session circumstances, as well as the characteristics of handlers, clients, and individual dogs—including age, experience, and gender—significantly influenced dogs’ responses. Specifically, older dogs were less playful but more settled, while experience was linked to positive affect in AAAs and AAT, but not in AAC. Female dogs demonstrated increased uncertainty and arousal in AAAs and AAE. The impact of session length varied by context. In AAC, unfamiliar handlers increased tension. Additionally, younger clients were associated with heightened uncertainty or tension in dogs across AAAs, AAC, and AAE. In light of these findings, optimising dog welfare requires matching dogs to suitable roles, attentive session planning, and managing workload.

## Simple Summary

This study examined the emotional experiences of 63 dogs across 837 animal-assisted service sessions, encompassing animal-assisted activities, education, coaching, and therapy. Behaviours were systematically recorded, and a Principal Component Analysis was performed to identify key affective states, including playfulness, comfort, anxiety, and uncertainty. Session circumstances, client and handler factors, and individual dog characteristics, like age, experience, and gender, significantly influenced dogs’ responses. This study highlights that animal-assisted services impact dogs differently depending on these factors, and positive welfare relies on carefully matching each dog to appropriate session types and designing sessions that support their well-being.

## 1. Introduction

Animal-assisted services (AASs), including therapy (AAT), education (AAE), coaching/counselling (AAC), and activities (AAAs), represent a rapidly expanding field in human healthcare, social work, and education [1,2].

Dogs and horses are among the most common species involved in AASs, with dogs now frequently included in psychological and educational interventions in Europe and North America [3,4,5]. In the Netherlands, AASs are offered in various settings, such as schools, mental health services, care facilities, and private coaching practices, with an estimated thousands of sessions conducted annually [6]. This increase has led to calls to improve professionals’ skills, enhance welfare monitoring, and strengthen the ethical justification for using animals.

Over the past few decades, the expanding literature on AASs has indicated beneficial effects on emotional regulation, social engagement, and cognitive functioning, especially in vulnerable populations such as children, older adults, and individuals with developmental or psychosocial challenges [7,8,9]. In autism spectrum disorder (ASD), dog- and horse-assisted services have most consistently been linked to increased social interaction, although evidence for long-term psychological or physiological effects remains limited [10,11,12]. Short-term reductions in human cortisol levels have been observed during dog-assisted sessions, especially when sessions last longer than 15 min, suggesting a potential dose-dependent stress-buffering effect [13]. Despite these promising findings, the evidence remains inconsistent due to small sample sizes, varied intervention models, and limited experimental controls, which affect generalizability [14,15]. This points to the need for more rigorous, standardised research to clarify both the effectiveness and ethical sustainability of AASs [16,17,18]. Accordingly, the specific settings and demands of these services necessitate specialised expertise, with animal welfare constituting a central ethical and methodological concern [19].

Dogs are widely recognised as sentient beings capable of experiencing a full range of affective states, including fear, joy, frustration, comfort, and pain [20]. Recognising their sentience creates a moral responsibility to protect their welfare, as animal well-being directly affects the quality and safety of interventions. From a One Welfare perspective, it is essential that the human–animal relationship promotes the well-being of both parties, rather than prioritising one over the other [6,21]. Therefore, animal-assisted services must be designed to benefit both dogs and humans equally [1,16,22].

Research has shown that dogs participating in animal-assisted services (AASs) are exposed to a variety of situational demands and challenges that may affect their well-being [23]. Several studies have examined both physiological and behavioural indicators of welfare in these settings, yielding mixed results. For instance, some studies have reported no significant increase in stress-related markers during or following AAS sessions [24,25,26,27].

However, it is important to acknowledge that welfare outcomes can be influenced by multiple contextual and individual factors, such as transportation demands, familiarity with the environment, session frequency, and the dog’s age. For example, Ng et al. [28] found that dogs showed higher salivary cortisol levels in unfamiliar environments compared with familiar ones, and Haubenhofer and Kirchengast [29] observed greater physiological stress on working days than at home, and with a higher frequency of sessions. Silas, Binfet, and Ford [30] found that dogs, when observed in a home environment, exhibited increased signs of stress after sessions. Therefore, further research is necessary to clarify how these contextual and individual conditions may limit a dog’s comfort and well-being during AASs [31].

Additionally, existing reviews point to considerable methodological heterogeneity and a lack of standardised assessment tools [23,31], complicating cross-study comparisons. This lack of standardisation and comparability has been identified as a key limitation in the field, hindering the development of a robust evidence base. Together, these limitations contribute to ongoing uncertainty regarding when AAS constitute a “win–win” interaction and when participation may compromise canine well-being [32].

A recent consensus defines positive animal welfare as a state in which animals flourish, experience predominantly positive lives, and develop competence and resilience [33]. This perspective moves beyond simply preventing negative outcomes such as fear, hunger, or pain. Instead, it emphasises actively promoting enjoyable experiences, opportunities to engage in natural, meaningful behaviours, and the ability to feel positive emotions, while maintaining overall well-being. Reflecting this, current frameworks stress the importance of fostering positive affect [20,32,34]. Applied to AAS, this perspective underscores the importance of ensuring that dogs genuinely enjoy their work, which is increasingly recognised as a keystone of ethical practice [22]. However, in much of the existing animal-focused AAS literature, there remains a bias toward negative welfare indicators, with comparatively limited attention to positive affective states [31,35]

Dogs express their emotional state through coordinated postural, facial, and behavioural signals. Signals such as tense tail wagging, avoidance, panting, yawning, lip-licking, slowed movement, and displacement behaviours are typically associated with negative affect, including stress, fear, or uncertainty [25,36,37].

In contrast, play, affiliative behaviour, sniffing, and voluntary human-directed interaction are reported as indicators of positive affect and good welfare, although their operationalisation varies [38]. Play in adult dogs, expressed through play bows, playful vocalisations, and loose movements, reflects emotional security and has been shown to reduce stress and cortisol levels [39,40,41,42]. Affiliative behaviours, such as relaxed tail wagging, body rubbing, and social licking, further promote social bonding and are negatively associated with physiological stress [26,32]. Sniffing serves both information-gathering and emotional regulation, and stimulates dopaminergic reward pathways, making it a key contributor to positive emotional states [43,44]. Human–dog bonding further reinforces positive affect: mutual gazing increases oxytocin release in both species, strengthening interspecies attachment [45], while short latency to voluntarily approach a human indicates a positive human–animal relationship and curiosity [33]. Notably, behaviours remain context-dependent and must be interpreted in combination with posture and situational cues.

Despite the growing popularity of animal-assisted services (AASs), robust evidence of their impact on the welfare of participating dogs remains limited [23,46]. Research in this area faces challenges due to the complex and variable nature of AASs, in which outcomes are shaped by human–animal relationships and contextual factors [47]. Accordingly, welfare outcomes are influenced by dog characteristics, handler skills, session context, and environmental unpredictability [22,29,32,48]. However, much of the existing research is based on controlled or small-scale designs, limiting ecological validity and insight into how these interacting factors shape dogs’ experiences in real-world AAS practice [24,49].

Consequently, most welfare assessments rely on a combination of behavioural observation, physiological measures, and handler reports, each with notable limitations, and few studies examine how dogs actually experience AASs in real-world, large-scale settings [22,23,31]. Findings are often inconsistent, reflecting a lack of standardised welfare assessment protocols and a tendency to focus on negative rather than positive indicators of well-being [31,50]. Importantly, there remains a lack of studies that simultaneously capture both positive and negative affective states within the same analytical framework in real-world AAS contexts.

In this context, approaches such as Principal Component Analysis (PCA) allow co-occurring behaviours to be clustered into latent patterns that can be interpreted as affective states, providing a potentially useful tool for capturing the complexity of animal emotional experience during AASs, as reported in different contexts (e.g., [51,52]). Additionally, including not only behaviours related to negative affect but also positive welfare indicators provides insight into whether it can be beneficial and whether the animals enjoy it [53].

These challenges highlight the need to identify both positive and negative affective states in dogs participating in AASs and to examine how these states relate to specific dog-, professional-, and session-level variables. Building on previous work, this study combines multivariate behavioural analysis with real-world observational data to address key gaps in ecological validity and the integration of positive and negative welfare indicators. Accordingly, the present study aims to evaluate behavioural indicators of dogs’ affective states during animal-assisted service sessions using a multivariate approach, and to identify the dog-, professional-, client-, and context-related variables associated with these affective states.

## 2. Materials and Methods

### 2.1. Study Design Overview

This research was conducted in collaboration with the Dutch Dog-Assisted Services (DASs) sector and consisted of two components: (1) a field study and (2) an evaluation exercise to establish behavioural thresholds relevant to DASs. The results from the evaluation exercise are reported in [54]. The field study involved 30 DAS professionals and 63 dogs.

### 2.2. Ethical Statement

This research was carried out in alignment with the Declaration of Helsinki and received approval from the Data Protection Officer at Aeres University of Applied Sciences, Dronten. Ethical review and approval for animals were waived for this study in compliance with the directive and current Dutch laws, as the experiments involved only behavioural observations and non-invasive interactions with the dogs. The dogs involved in this research were not classified as research animals (protocol code: AER2025-09).

### 2.3. Study Design and Data Collection

Participants were recruited through the professional networks of project partners, supplemented by targeted social media advertisements and online informational webinars. All participants completed a structured online training focused on using the ethogram to identify and score dog behaviours observed during AAS sessions. A retrospective scoring approach, adapted from Visser et al. [53], was employed to reflect real-world practice, in which professionals typically evaluate dog behaviour after the session has concluded.

#### 2.3.1. Ethogram

A canine ethogram was developed based on expert consultation, a review of the scientific literature on canine emotion and welfare, and an analysis of 20 videotaped DAS sessions. These sessions involving dogs from seven different breeds were specifically recorded to ensure that the ethogram comprehensively captured behaviours relevant to AAS contexts. This iterative process resulted in a final set of 19 observable behaviours (Table 1). All video data were recorded using Sony FDR-AX53 cameras (Sony Corporation, Tokyo, Japan) and a Pixio camera tracking system (Move ‘N See, Vannes, France), with all recordings captured in high-definition (HD) quality. Client privacy was safeguarded through informed consent, and participants retained the right to withdraw consent for use of the recordings at any time.

For each behaviour, operational definitions were developed based on the peer-reviewed literature to ensure consistent identification and scoring across observers. These definitions were applied throughout this study and may differ from informal interpretations commonly used in professional practice. The ethogram, as seen in Table 1, included behaviours indicative of both negative and positive affective states. Each of the 19 behaviours was scored using a three-level categorical scale: absent, occasionally, and frequently. Thresholds distinguishing “occasionally” from “frequently” were defined a priori and differed by behaviour. These thresholds were calibrated using a subsample of the recorded video material, combined with expert judgement and evidence from the existing literature. Video recordings were systematically reviewed to determine realistic frequency counts and/or proportions of session duration for each behaviour, ensuring that category boundaries reflected meaningful behavioural differences. For example, a broad, slow tail wag was classified as “occasionally” when present for 1–25% of the session duration and “frequently” when present for more than 25%, whereas avoidance or backing away was classified as “occasionally” when observed once or twice and as “frequently” when observed more than twice within a session (Table 1).

#### 2.3.2. Training

Participation required completion of a mandatory online training programme provided and delivered by the authors. A subset of the previously recorded sessions was used to extract short video clips illustrating the 19 selected behaviours. During the training, professionals were instructed to recognise and score these behaviours using the predefined operational definitions and a digital app. Competency was assessed through a video-based test administered in Test Vision (Teelen, Wilp, The Netherlands; https://testvision.nl), with a minimum passing score of 75%, consistent with the training procedures used for equine-assisted professionals described by Visser et al. [53]. The training programme, including the competency test, required an average of approximately 6 h per participant. A total of five online training sessions were delivered between October 2023 and January 2024.

#### 2.3.3. Collection of Data

The data collection method was adapted from the methodology described by Visser et al. [53]. Data were collected using a custom-built digital scoring application developed in Microsoft Power Apps (Microsoft Corporation, Redmond, WA, USA), version 23081, which enabled real-time data entry on multiple devices (smartphone and laptop), secure institutional data storage, and post-session editing of records. Only trained participants were granted access to the application environment. Upon submission, the data were automatically synchronised and exported for subsequent statistical analysis. Each participant monitored one or more dogs during each AAS session over a two-month observation period. Data collection took place between October 2023 and April 2024.

Following each session, handlers completed a questionnaire within the application. In addition to behavioural data, contextual information was collected, including session duration, activity type, use of materials or food, familiarity between the dog, professional, and client, environmental conditions, transport characteristics, number of people and animals present, leash use, previous workload, notable events, and the handler’s perceived session load for the dog. Client-related variables included age, session number, familiarity with the dog, and referral pathway.

Each participating handler completed a one-time questionnaire capturing demographic and handler characteristics, including age, gender, education level, professional training, and years of experience. These data were linked to session-level data via participant identifiers.

Data on dog characteristics were collected once for each of the participating dogs and included information on living situation, management practices, health status, age, and years of work experience. Breed information was recoded into ten Fédération Cynologique Internationale (FCI) breed groups for analytical purposes. Canine personality was assessed using the Dog Personality Questionnaire (DPQ) developed by Jones [79]. The DPQ comprises 45 items rated on a 7-point Likert scale and assesses five personality dimensions: fearfulness, aggression toward people, activity/excitability, responsiveness to training, and aggression toward animals. Scores were converted to percentages of the maximum possible score per dimension, and composite personality profiles were calculated for each dog.

### 2.4. Data Analysis

Initially, 71 dogs were enrolled in this study. Dogs were included in the analysis if they were monitored over a complete 2-month period and had completed both the dog characteristics and the handler questionnaire. In total, 63 dogs (26 males and 37 females) from 30 handlers contributed to 837 sessions included in the analysis. Dogs that did not complete any sessions (*n* = 8) were excluded from further analysis.

Descriptive statistics were used to summarise session characteristics and behavioural frequencies.

Principal Component Analysis (PCA) was conducted separately for each AAS category (AAA, AAC, AAE, and AAT) to identify clusters of co-occurring behaviours representing underlying affective components. Sampling adequacy was assessed using the Kaiser–Meyer–Olkin (KMO) measure. The number of components was determined using the Kaiser criterion (eigenvalues > 1) and visual inspection of the scree plot to identify the “elbow.” An orthogonal rotation was applied to enhance interpretability. The interpretation and labelling of components were guided by the behavioural variable loadings and informed by consultation with field experts.

Subsequently, multiple linear regression analyses were performed to examine which dog-related, session-related, and handler-related variables significantly contributed to the extracted PCA components.

All analyses were performed using Microsoft Excel 365 (Microsoft Corporation, Redmond, WA, USA) and the statistical programs IBM SPSS Statistics (IBM Corporation, Armonk, NY, USA), version 28.0 and R (R Foundation for Statistical Computing, Vienna, Austria), version 4.4.0. For all analyses, values of *p* < 0.05 were considered statistically significant.

## 3. Results

### 3.1. Session Characteristics

Of the 837 sessions, the largest proportion consisted of animal-assisted therapy (AAT, *n* = 316), followed by animal-assisted activities (AAAs, *n* = 255) and animal-assisted coaching (AAC, *n* = 195). The fewest sessions were observed in animal-assisted education (AAE, *n* = 71). The average session duration varied across categories. AAT sessions were the longest, with a mean duration of 48 min, followed by AAC (45 min) and AAAs (35 min). AAE sessions were the shortest, with an average duration of 25 min.

**Figure 1:**
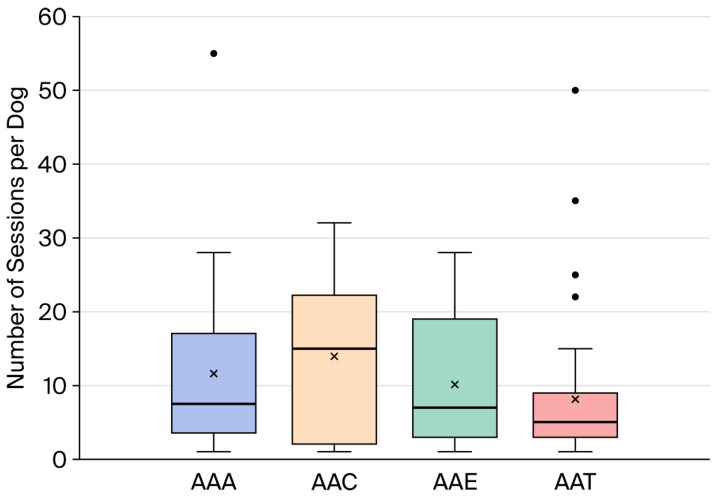
Distribution of session frequency per dog over a two-month period, stratified by category. The categories include animal-assisted activities (AAAs), animal-assisted coaching (AAC), animal-assisted education (AAE), and animal-assisted therapy (AAT). The boxplots display the median (horizontal line), interquartile range (box and whiskers), and outliers (dots). Mean scores are marked with an X.

There was substantial variation in the number of sessions in which dogs participated during the two-month study period. Session frequency per dog varied across AAS types. AAC showed the highest mean session frequency (1.74 sessions per week; median = 1.88), followed by AAAs (mean = 1.45; median = 0.94) and AAE (mean = 1.27; median = 0.88). AAT exhibited the lowest session frequency (mean = 1.01; median = 0.63) (see Figure 1). Additional details on the session characteristics are provided in Supplementary Table S1.

### 3.2. Handler Characteristics

A total of 30 handlers participated in this study. One handler did not complete the questionnaire on professional characteristics, resulting in available demographic data for 29 observers. Of these, 27 identified as female, and two as male. The majority of these professionals (77%) were between 31 and 60 years of age; one participant was older than 60, and the remaining participants were younger than 31.

Not all handlers listed their years of professional experience. Among those who did, more than half had three years or less in animal-assisted work. Experience ranged from less than 1 year to 15 years. The majority of handlers worked with their own dogs (70%). A smaller proportion worked exclusively with dogs they did not own (13%), while 14% worked with both their own dog and dogs owned by others. Some of the non-owned dogs were housed with foster families, although this was not the case for all of them. Additional details on the handler characteristics are provided in Supplementary Table S2.

### 3.3. Client Characteristics

**Figure 2:**
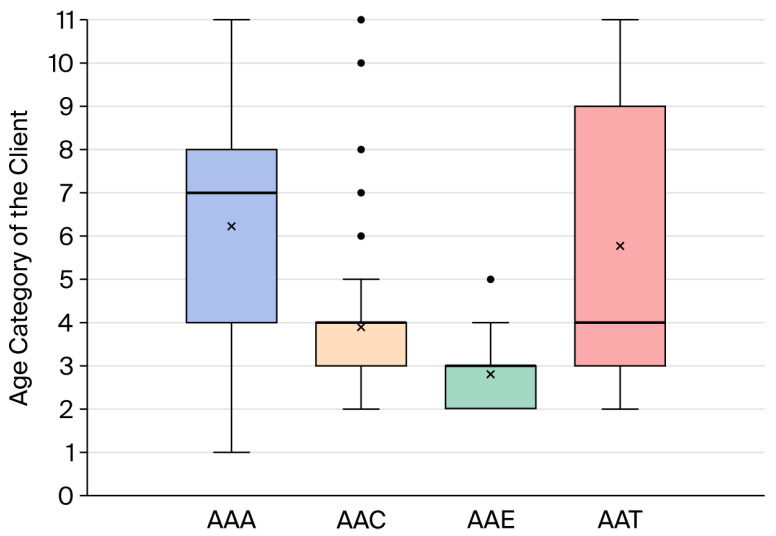
Distribution of age score in age category for clients in AAAs, AAC, AAT, and AAE sessions. Age categories were coded as follows: 1 = 0–4 years, 2 = 4–8 years, 3 = 8–12 years, 4 = 12–16 years, 5 = 16–18 years, 6 = 18–25 years, 7 = 25–30 years, 8 = 30–40 years, 9 = 40–50 years, 10 = 50–60 years, and 11 = >60 years. The boxplots display the median (horizontal line), interquartile range (box and whiskers), and outliers (dots). Mean scores are marked with an X.

Client age distributions differed across AAS types, with animal-assisted therapy (AAT) and animal-assisted activities (AAAs) involving a broader and generally older age range, whereas animal-assisted coaching (AAC) and animal-assisted education (AAE) were more frequently delivered to younger clients (see Figure 2).

### 3.4. Dog Characteristics

This study included 63 dogs (26 males and 37 females) monitored over a two-month period. The mean age was 5.01 years (SD = 2.6; range: 1 to 11 years), with most dogs aged 2–5 years and a relatively stable representation of dogs aged 6–9 years. Approximately half of the dogs began working at ≥2 years of age, and 10 dogs were younger than 1 year, while the remainder started at ≤2 years of age. Most dogs had no diagnosed medical conditions; nine dogs were reported to have health issues, primarily orthopaedic disorders or allergies.

A diverse range of breeds was represented, with FCI group retrievers, spaniels, and water dogs forming the largest groups. Based on the Dog Personality Questionnaire, dogs showed high mean scores for responsiveness to trainability (79% ± 12) and activity/excitability (75% ± 8). Lower scores were observed for aggression toward animals (36% ± 11) and fearfulness (32% ± 11). Additional details on the dog characteristics are provided in Supplementary Table S3.

### 3.5. Dogs’ Behaviour

The behaviour of the dogs was evaluated for 19 specific behaviours after each session (see Table 1).

The extent and intensity of interactions with clients and handlers varied notably across the different AAS categories. While client interaction was observed in 52% of AAA sessions, it was a constant feature in all AAC, AAE, and AAT sessions. The highest intensity of client interaction—defined as occurring for more than 25% of the session duration—was recorded in AAC (78%) and AAT (57%). Interaction with the handler was nearly universal in AAC, AAE (100%), and AAT (99%), though it was predominantly scored as ‘occasional’ (1–25% of the duration). In contrast, handler interaction in AAA sessions occurred in 30% of cases.

Specific behavioural expressions also showed context-dependent patterns. For instance, wide, slow tail wagging was highly prevalent in AAA sessions (90%) but was observed significantly less frequently in AAE (41%). Lying down occurred most often in AAE (83% of sessions) and AAT, while being least prevalent in AAAs. Lip licking was recorded in approximately half of the AAA (53%) and AAC (54%) sessions, occurring almost exclusively at an ‘occasional’ level. Other behaviours, including sniffing the ground, play behaviour, body shaking, and self-grooming, were regularly observed in AAC and AAE, though these were more commonly scored as occasional rather than frequent. Detailed percentages for all 19 recorded behaviours across the different AAS types are provided in the Supplementary Materials (Tables S4–S7).

### 3.6. Affective States

Principal Component Analysis (PCA) was conducted separately for each AAS category to identify clusters of behaviours representing affective states. For ease of interpretation, component scores were rescaled to a 1–10 scale, with 1 indicating ‘not at all’ and 10 indicating ‘all the time’.

#### 3.6.1. AAA Sessions

A Principal Component Analysis (PCA) identified four components explaining 45.1% of the total variance (KMO = 0.656). Based on behavioural loadings, the components were labelled ‘detached’, ‘thoughtful’, ‘uncertain’, and ‘playful’ (see Table 2).

**Figure 3:**
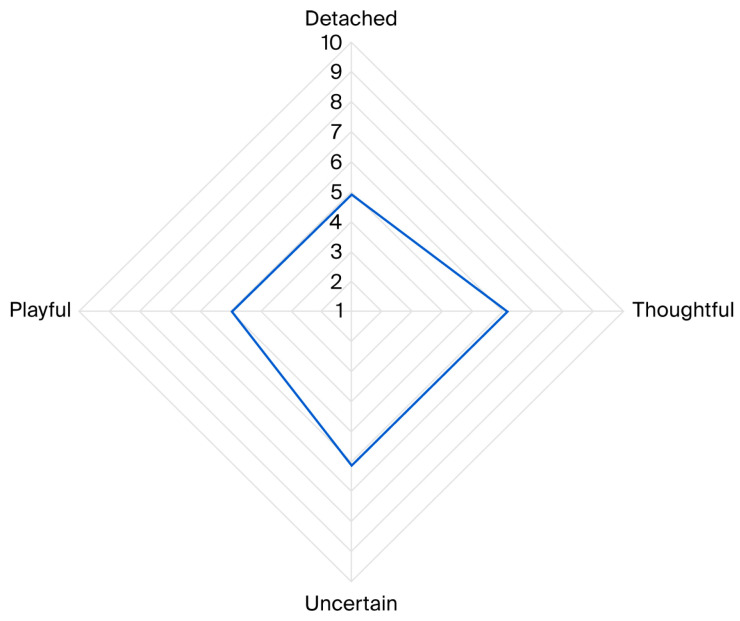
Radar chart illustrating average scores for affective states displayed by the participating dogs during 255 AAA sessions, including detached, thoughtful, uncertain and playful. Scores ranged from 1 to 10, with higher values indicating a stronger presence of the corresponding state.

The average scores (STDEV) for the components were ‘detached’: 4.90 (1.49), ‘thoughtful’: 6.16 (1.17), ‘uncertain’: 6.13 (1.42), and ‘playful’: 4.96 (1.38). The affected state labelled as ‘thoughtful’, here marked by behaviours such as interaction with the client and broad, slow tail-wagging, was the most predominantly displayed state by dogs during AAA sessions, followed by ‘uncertain’. This state was characterised by behaviours like body shaking and interaction with the handler. ‘Playful’ and ‘detached’ states were less commonly observed, with the latter identified through behaviours such as sniffing the ground and being out of sight (see Figure 3).

#### 3.6.2. AAC Sessions

A Principal Component Analysis (PCA) was conducted using 16 behavioural variables. Five components were identified, explaining 61.2% of the variance (KMO = 0.729). Based on behavioural loadings, the components were labelled ‘playful’, ‘comfortable’, ‘tense’, ‘anxious’, and ‘uncertain’ (see Table 3).

**Figure 4:**
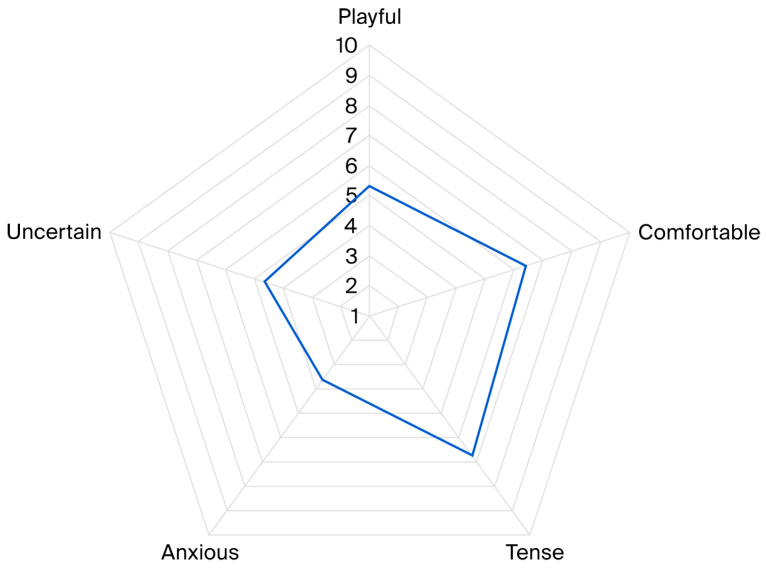
Radar chart illustrating average scores for affective states displayed by the participating dogs during 195 AAC sessions, including playful, comfortable, tense, anxious, and uncertain. Scores ranged from 1 to 10, with higher values indicating a stronger presence of the corresponding state.

The mean (STDEV) component scores were as follows: ‘playful’: 5.32 (1.64), ‘comfortable’: 6.40 (1.82), ‘tense’: 6.74 (1.61), ‘anxious’: 3.64 (1.09), and ‘uncertain’: 4.64 (1.45). During AAC sessions, dogs most commonly showed the affective state labelled as ‘tense’, characterised by behaviours such as lip licking and vocalisation. The ‘comfortable’ affective state, identified through behaviours such as broad, slow tail wagging, lying down, and self-grooming, was the second most prevalent affective state observed during AAC sessions, followed by the states ‘playful’, ‘uncertain’, and ‘anxious’ (see Figure 4).

#### 3.6.3. AAE Sessions

A Principal Component Analysis (PCA) was conducted with 17 behaviour variables. Four components were identified, explaining 52.4% of the variance (KMO = 0.587). Based on behavioural loadings, the components were labelled ‘engaged’, ‘uncertain’, ‘anxious’, and ‘aroused’ (see Table 4).

**Figure 5:**
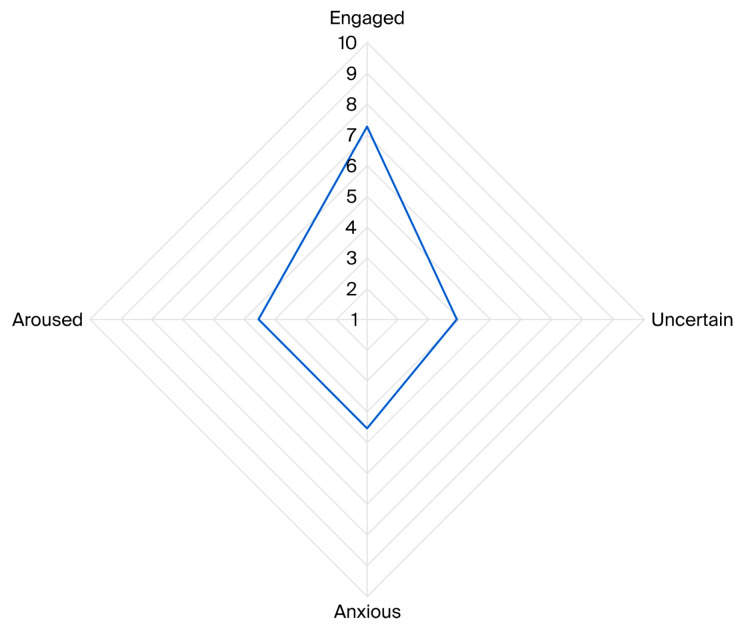
Radar chart illustrating average scores for affective states displayed by the participating dogs during 71 AAE sessions, including engaged, uncertain, anxious, and aroused. Scores ranged from 1 to 10, with higher values indicating a stronger presence of the corresponding state.

The mean (STDEV) scores for the components were as follows: ‘engaged’: 7.27 (2.00), ‘uncertain’: 3.91 (1.14), ‘anxious’: 4.54 (1.86), and ‘aroused’: 4.53 (2.21). During AAE sessions, dogs most commonly displayed the affective state labelled as ‘engaged’, characterised by interaction with the client and the display of broad, slow tail wagging, in the absence of play behaviour, followed by anxious states, marked by behaviours such as interaction with the handler and body shaking, and aroused and uncertain states (see Figure 5).

#### 3.6.4. AAT Sessions

A Principal Component Analysis (PCA) was conducted using 16 behavioural variables. Four components were identified, explaining 49.7% of the variance (KMO = 0.723). Based on the behavioural loadings, the components were labelled ‘enthusiastic’, ‘anxious’, ‘release tension’, and ‘playful’ (see Table 5).

**Figure 6:**
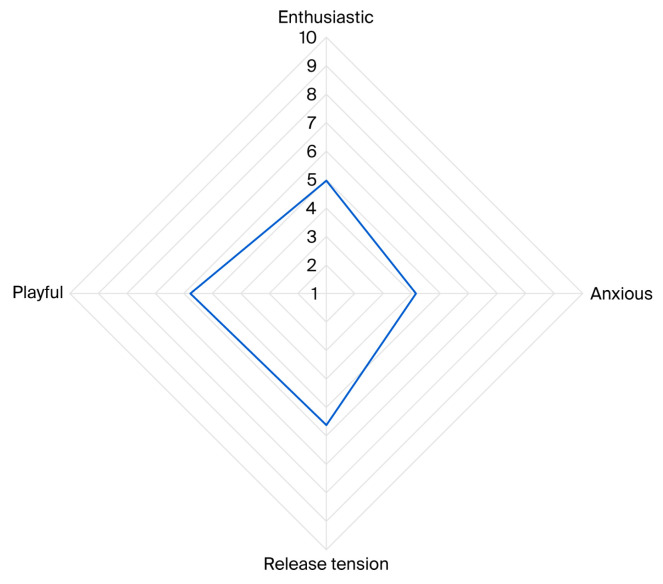
Radar chart illustrating average scores for affective states displayed by the participating dogs during 316 AAT sessions, including enthusiastic, anxious, release tension, and playful. Scores ranged from 1 to 10, with higher values indicating a stronger presence of the corresponding state.

The mean (STDEV) scores for the components were ‘enthusiastic’: 4.97 (2.06), ‘anxious’: 4.15 (1.74), ‘release tension’: 5.62 (1.47), and ‘playful’: 5.77 (1.46). The affective state ‘playful’, characterised by play behaviour and interaction with the handler, was the most frequently observed state during AAT sessions. Following this, the affective state ‘release tension’ was the second most prevalent, identified through behaviours such as yawning and self-grooming. Next were enthusiastic states, characterised by high, fast tail wagging, high posture, and client interaction, and anxious states, marked by avoidance, lip licking, delayed movements, and self-grooming (see Figure 6).

### 3.7. Associations Between Affective States and Explanatory Variables by AAS Type

#### 3.7.1. AAA Sessions

For AAAs, no association with session frequency was found. However, a longer session duration was associated with higher scores on the ‘detached’ (B = 0.04, SE = 0.01, and *p* < 0.001) and ‘uncertain’ components (B = 0.02, SE = 0.01, and *p* < 0.05). Previous work earlier in the day (e.g., day-care context) was also associated with higher scores on the ’detached’ component (B = 0.08, SE = 0.04, and *p* < 0.05). Conversely, when dogs had not worked earlier that day, scores on the ‘detached’ component were lower (B = −0.05, SE = 0.02, and *p* < 0.01), and scores on the ‘uncertain’ component were higher (B = 0.06, SE = 0.02, and *p* < 0.01).

Client age was negatively associated with scores on the ‘detached’ component (B = −0.01, SE = 0.00, and *p* < 0.01), the ’uncertain component (B = −0.01, SE = 0.00, and *p* < 0.001), and the ‘playful’ component (B = −0.01, SE = 0.00, and *p* < 0.01). Client unfamiliarity with the dog was positively associated with scores on the ‘playful’ component (B = 0.07, SE = 0.02, and *p* < 0.01).

Dog-related variables showed that higher dog age was associated with increased scores on the ‘detached’ component (B = 0.01, SE = 0.00, and *p* < 0.001). A later starting age in AASs was associated with lower scores on the ‘playful’ component (B = −0.18, SE = 0.04, and *p* < 0.001). In addition, greater years of experience were associated with higher scores on the ‘thoughtful’ (B = 0.19, SE = 0.04, and *p* < 0.001) and ‘playful’ components (B = 0.08, SE = 0.02, and *p* < 0.001), and with lower scores on the ‘uncertain’ component (B = −0.06, SE = 0.01, and *p* < 0.001). Breed-group differences were observed (relative to cross-breeds). For example, Sheepdogs and Cattledogs showed higher scores on the ‘cautious’ component (B = 0.20, SE = 0.03, and *p* < 0.001) compared with cross-breeds, as did the Retrievers–Flushing Dogs–Water Dogs group (B = 0.17, SE = 0.05, and *p* < 0.01). Sex effects indicated that female dogs scored lower on the ‘thoughtful’ (B = −0.34, SE = 0.07, and *p* < 0.001) and ‘playful’ components (B = −0.11, SE = 0.02, and *p* < 0.001) and higher on the ‘uncertain’ component (B = 0.10, SE = 0.02, and *p* < 0.001) compared with male dogs. Finally, the absence of orthopaedic conditions was associated with higher ‘thoughtful’ (B = 0.20, SE = 0.05, and *p* < 0.001) and ‘playful’ scores (B = 0.22, SE = 0.06, and *p* < 0.001) alongside lower ‘uncertain’ scores (B = −0.24, SE = 0.04, and *p* < 0.001).

No significant effects were found for the handler’s education level or years of experience. An overview of all other significant associations for AAAs is provided in Supplementary Table S8.

#### 3.7.2. AAC Sessions

For AAC, no associations were found with session frequency. In contrast, a longer session duration was associated with higher scores on the ‘playful’ component (B = 0.04, SE = 0.01, and *p* < 0.001). Dogs that had not worked earlier that day exhibited higher scores on the ‘anxious’ component (B = 0.04, SE = 0.02, and *p* < 0.05).

Client age was negatively associated with scores on the ‘tense’ component (B = −0.04, SE = 0.01, and *p* < 0.001), and greater client familiarity was associated with lower scores on the ‘playful’ (B = 0.05, SE = 0.02, and *p*< 0.01) and ‘comfortable’ components (B = 0.06, SE = 0.03, and *p* < 0.05).

Older dogs showed lower scores on the ‘playful’ (B = −0.07, SE = 0.01, and *p* < 0.001) and ‘uncertain’ components (B = −0.11, SE = 0.02, and *p* < 0.001), but higher scores on the ‘comfortable’ (B = 0.24, SE = 0.02, and *p* < 0.001) and ‘anxious’ components (B = 0.09, SE = 0.02, and *p* < 0.001). In addition, greater years of experience were associated with higher scores on the ‘tense’ component (B = 0.04, SE = 0.01, and *p* < 0.001).

Handler-related variables were largely non-significant; however, handler unfamiliarity with the dog was associated with higher scores for the ‘tense’ component (B = 0.53, SE = 0.14, and *p* < 0.001). An overview of all other significant associations for AAC is provided in Supplementary Table S9.

#### 3.7.3. AAE Sessions

No associations were found with mean weekly session frequency. However, a longer session duration was associated with lower scores on the ‘uncertain’ component (B = −0.17, SE = 0.07, and *p* < 0.05).

Client age was associated with lower scores on the ‘uncertainty’ component (B = −0.07, SE = 0.03, and *p* < 0.05) and higher scores on the ‘arousal’ component (B = 0.13, SE = 0.05, and *p* < 0.05), while client familiarity with the dog was associated with higher scores on the ‘uncertain’ component (B = −0.13, SE = 0.06, and *p* < 0.05).

Older dogs exhibited lower scores on the ‘engaged’ component (B = −0.54, SE = 0.14, and *p* < 0.001) and the ‘anxious’ component (B = −0.68, SE = 0.10, and *p* < 0.001), but higher scores on the ‘uncertain’ component (B = 0.91, SE = 0.19, and *p* < 0.001). Female dogs showed lower scores on the ‘engaged’ (B = −3.07, SE = 0.97, and *p* < 0.01) and ‘anxious’ components (B = −4.67, SE = 0.67, and *p* < 0.001), alongside higher scores on the ‘uncertain’ (B = 5.34, SE = 1.02, and *p* < 0.001) and ‘aroused’ components (B = 0.51, SE = 0.10, and *p* < 0.001) scores. Personality traits and health conditions were not associated with affective states in AAE.

Handlers’ background variables also showed no significant associations. An overview of all other significant associations for AAE, including breed-related effects, is provided in Supplementary Table S10.

#### 3.7.4. AAT Sessions

A higher mean weekly session frequency was associated with lower scores on the ‘enthusiastic’ component (B = 0.07, SE = 0.01, and *p* < 0.001), whereas session duration was not significantly associated with affective states. Prior work attendance at a day-care setting earlier in the day was associated with higher scores on the ‘release tension’ component (B = 0.26, SE = 0.07, and *p* < 0.001).

Client-related variables were not significantly associated with affective states in AAT.

Several dog-level variables were associated with affective states: higher dog age was associated with lower scores on the ‘enthusiastic’ component (B = −0.03, SE = 0.01, and *p* < 0.01), greater years of experience was associated with higher scores on the ‘enthusiastic’ (B = 0.04, SE = 0.01, and *p* < 0.01) and ‘playful’ components (B = 0.02, SE = 0.01, and *p* < 0.05); and a later starting age in AAT was associated with higher scores on the ‘enthusiastic’ (B = 0.13, SE = 0.03, and *p* < 0.001), ‘anxious’ (B = 0.05, SE = 0.02, and *p* < 0.01), and ‘playful’ components (B = 0.07, SE = 0.02, and *p* < 0.001). Personality traits were also associated with affective states in AAT, with higher fearfulness associated with higher scores for ‘anxious’ (B = 0.01, SE = 0.00, and *p* < 0.001) and ‘playful’ (B = 0.01, SE = 0.00, and *p* < 0.001) components, and higher excitability/activity was associated with higher scores for the ‘playful’ component (B = 0.01, SE = 0.00, and *p* < 0.01).

Living situation was likewise associated with affective states: dogs living in foster families (as opposed to living with the handler) showed lower scores on the ‘enthusiastic’ (B = −0.25, SE = 0.03, and *p* < 0.001), ‘anxious’ (B = −0.11, SE = 0.04, and *p* < 0.01), and ‘playful’ components (B = −0.15, SE = 0.04, and *p* < 0.001) and higher scores for the ‘release tension’ component (B = 0.16, SE = 0.03, and *p* < 0.001). An overview of all other significant associations for AAT, including handler-related effects, is provided in Supplementary Table S11.

## 4. Discussion

### 4.1. Session Structure and Workload

The observed differences in session duration and workload across intervention types highlight the heterogeneity of animal-assisted services and underscore the importance of considering cumulative exposure when evaluating canine welfare. Although session length in this study was consistent with ranges reported in previous AAS research, substantial variation in session frequency and total workload per dog was evident, particularly in AAAs and AAC (Figure 1).

Such differences suggest that dogs in certain AAS contexts may experience considerably higher cumulative workloads than others. Given earlier findings linking higher workload to increased physiological stress in dogs in AAS [29], these patterns warrant careful consideration when interpreting welfare outcomes and developing guidelines for responsible AAS practice.

### 4.2. Handler and Dog Characteristics

The professional sample in this study was predominantly female and largely comprised individuals in mid-adulthood, a pattern consistent with earlier findings in animal-assisted services [80]. The wide variation in professional experience, together with differences in dog ownership and living arrangements, underscores the diversity of the practical contexts in which AASs are delivered. Such variability may influence how dogs experience their work, as factors such as handler familiarity, consistency of handling, and living arrangements have been suggested to affect canine stress and coping activity. These characteristics should, therefore, be considered when interpreting welfare outcomes and when developing training standards and professional guidelines. In addition, individual characteristics may influence how canine behaviour is perceived; for example, women have been found to report higher levels of empathy toward animals and may be more attentive to subtle behavioural cues [81]. While this does not necessarily compromise the quality of observations, it may contribute to variation in how dogs’ affective states are interpreted.

The dog sample in this study reflects a relatively young, actively working AAS population, with an age distribution comparable to those reported in earlier studies (e.g., [80]). The inclusion of dogs that started working at an early age, including some under one year, highlights ongoing variation in professional practices regarding the onset of AAS participation, which remains an area of ethical and welfare-related debate.

Health conditions were reported for a small subset of dogs, primarily orthopaedic issues and allergies. Since health status is infrequently reported in AAS studies [82], the lack of comparative prevalence data limits interpretation but underscores the importance of more systematic health reporting in future studies. Breed representation was diverse, with retrievers and crossbreeds predominating, consistent with previous AAS samples [80,83].

Personality profiles indicated high trainability and activity levels alongside low fearfulness and aggression, supporting previous characteristics of AAS dogs as socially orientated and emotionally stable [4,84]. While these traits are often considered desirable for AAS work, their interaction with workload, context, and management factors warrants careful consideration when evaluating long-term welfare outcomes.

### 4.3. Behavioural Responses

Behavioural patterns across the four AAS types provided important context for interpreting the PCA-derived affective states and highlighted how behavioural expression varied with service characteristics.

While interaction with clients and handlers was observed across all AAS types, the intensity and form of these services differed, reflecting the varying goals and structures of the interventions. More sustained client interaction in AAC aligns with the central role of human–dog cooperation in these service types [4], whereas more intermittent interaction in AAAs and AAE may reflect a less intensive or more educational nature.

Several behaviours commonly interpreted as indicators of relaxation or regulation—such as broad, slow tail wagging, lying down, play behaviour, and ground sniffing—were observed across intervention types but showed clear contextual variation. Broad, slow tail wagging, often associated with relaxed or affiliative states [85,86], occurred less frequently in AAE, suggesting that educational settings may evoke different affective or regulatory responses than therapeutic or coaching contexts. In contrast, low, tight, and fast tail wagging was more prevalent in AAC, potentially reflecting higher arousal levels in coaching-oriented interactions.

Additional behavioural differences further underscore context specificity: lip licking was more common in AAAs and AAC than in AAE and AAT, whereas body shaking occurred most frequently in AAE. Panting was least frequently observed in AAE, while lying down occurred least often in AAAs. Behaviour such as lip licking and ground sniffing, which have been described as calming or stress-regulating signals [37,87], were regularly observed but typically low-intensity, underscoring the importance of considering frequency and context rather than presence alone. Overall, these behavioural patterns emphasise that dogs’ responses in AASs are shaped by both individual characteristics and situational demands, supporting the need for context-sensitive interpretation of behaviour when assessing canine welfare in applied settings.

### 4.4. Affective States

The PCA-derived affective states further illustrate that dogs’ emotional experiences in AASs are both context-dependent and multi-dimensional. As noted in previous work, interpreting individual behaviours in isolation is problematic, as many behaviours can serve multiple emotional or regulatory functions depending on context [36,53,88,89]. Several affective states emerged across multiple AAS types, albeit with partially different behavioural compositions, underscoring that similar labels may reflect distinct underlying processes depending on the intervention context.

For example, the state labelled uncertain, observed across AAAs, AAC, and AAE, consistently involved behaviours such as body shaking and interaction with the handler, while lip licking contributed to this state in some but not all contexts (Table 2, Table 3 and Table 4). Behaviours such as lip licking and body shaking have previously been described as regulatory or stress-related responses rather than unambiguous indicators of negative affect [36,88,89], suggesting that uncertainty in AAS dogs may manifest through a flexible constellation of coping behaviours rather than a single behavioural marker.

Similarly, playful states were identified across multiple AAS types but were characterised by different combinations of behaviours. While play behaviour itself is commonly interpreted as an indicator of positive social engagement and well-being [40,90], accompanying behaviours such as elevated posture, panting, or avoidance varied between intervention types. This supports the view that play in applied AAS contexts may co-occur with heightened arousal or regulatory behaviours, rather than representing a uniformly relaxed affective state.

Other affective states appeared to be more context-specific. Detached and thoughtful states were unique to AAAs and were associated with behaviours such as sniffing the ground, temporary disengagement, lying down, and slower movement patterns. Ground sniffing and disengagement have been described as potential calming or self-regulatory strategies [88], suggesting that these states may reflect opportunities for autonomy or reduced task demands within activity-based sessions. In contrast, comfortable and tense states emerged only in AAC, potentially reflecting the structured and goal-oriented nature of coaching sessions, in which affiliative engagement and performance-related demands may alternate.

Finally, affective states identified in AAE and AAT further illustrate how similar behavioural elements may take on different meanings depending on interactional context (Table 4 and Table 5). In AAE, engaged and aroused states combined behaviours related to play, posture, and arousal regulation, whereas in AAT, sustained client interaction combined with high posture and tail-wagging behaviours supported the interpretation of a distinct enthusiastic state rather than arousal alone. The identification of a release tension state in AAT—characterised by yawning, grooming, body shaking, and lying down—aligns with descriptions of post-activation regulatory behaviours reported in the canine stress and recovery literature [36,89].

Taken together, these findings highlight that affective states in AASs should not be interpreted as fixed emotional categories, but as dynamic patterns of behavioural co-occurrence shaped by task demands, interactional roles, and environmental context. This reinforces the value of multivariate, context-sensitive approaches when assessing canine welfare in applied intervention settings [51,52].

These differences in frequency of behaviours suggest that each AAS type has its own range of behaviours. However, interpreting individual behaviours in isolation can be problematic, as many behaviours serve multiple emotional functions depending on the context [36,53,88,89]. Therefore, PCA was employed to cluster co-occurring behaviours within each AAS session to identify affective states.

### 4.5. Session-Related Associations with Affective State

The relationship between session duration and dogs’ affective states varied across AAS types, highlighting that longer or shorter sessions are not inherently beneficial or detrimental. Rather, how the dog experiences session length appears to depend on the intervention context and accompanying demands. In AAAs, longer sessions were associated with more ‘detached’ and ‘uncertain’ affective profiles, suggesting increased behavioural withdrawal or regulatory responding during prolonged exposure. Behaviours such as body shaking and lip licking, which have been linked to elevated cortisol levels [26], should not necessarily be interpreted as indicators of negative welfare alone, as they may also function as coping or stress regulation strategies in challenging situations [86]. Increased interaction with the handler in these contexts may similarly reflect support-seeking behaviour rather than distress per se [91,92].

In contrast, longer sessions in AAE were associated with lower uncertainty, suggesting that extended exposure in educational settings may allow dogs to settle and adapt, potentially due to more predictable environments or interaction patterns. Although the AAE sample was smaller, this finding points to the possibility that certain contexts are more conducive to calming and regulation over time. In AAC, longer sessions were associated with higher playful states, which may indicate that coaching environments provide sufficient safety and engagement for dogs to express play behaviour, a pattern previously linked to positive affective experiences in AAS contexts [32].

Session duration was not associated with affective states in AAT; however, higher weekly session frequency was linked to lower enthusiastic scores. This finding aligns with earlier work suggesting that cumulative workload, rather than single-session characteristics, may reduce positive engagement over time [29]. Together, these patterns underscore the importance of considering both session structure and cumulative exposure when evaluating dogs’ affective experiences in AASs.

### 4.6. Dog-Related Associations with Affective State

Dog age and experience were consistently associated with affective patterns across AAS types, underscoring their relevance for interpreting dogs’ emotional responses in applied settings. Across intervention types, increasing age was generally associated with lower engagement and playfulness and with more settled or uncertain affective profiles. This aligns with broader behavioural research indicating that younger dogs tend to show high energy, sociability, and attachment-seeking behaviour, whereas older dogs display reduced affiliative engagement and activity alongside greater behavioural calm and task focus [80,93,94]. In educational and coaching contexts, older dogs appeared more settled yet also showed higher uncertainty or anxiety-related states, suggesting that reduced behavioural expressiveness does not necessarily equate to uniformly positive affect. Previous work has shown that older therapy dogs may display more regulatory or affiliative behaviours while exhibiting lower physiological stress markers, indicating that such behaviours may function as calming or coping strategies rather than signs of diminished welfare [26]. Conversely, other studies suggest that increased experience may reduce stress responsiveness through habituation [73], highlighting that age-related effects likely reflect a balance between habituation, changing motivation, and coping style.

Experience-related associations further emphasised this complexity. In AAAs and AAT, greater experience was associated with more positive or engaged affective states, suggesting improved coping and predictability over time. In contrast, in AAC, greater experience was linked to higher tense states, consistent with findings that repeated exposure in certain working contexts may increase cumulative stress load [29,31]. Associations with starting age similarly suggest that early work experiences may shape later affective responses, although empirical evidence on optimal starting ages in AASs remains limited [31].

Sex-related patterns were context-specific. Female dogs showed more uncertain and aroused profiles and lower engagement or playfulness in AAAs and AAE, consistent with previous findings that females tend to score higher on insecurity and human-directed sociability, while males show higher energy and play motivation [93,94,95,96]. The absence of sex differences in AAT and AAC suggests that task structure and interactional demands may moderate these effects.

### 4.7. Handler-Related Associations with Affective State

Handler-related characteristics were associated with dogs’ affective states in specific AAS contexts, highlighting the importance of the human–dog relationship in shaping emotional responses during sessions. In AAT, higher professional age was associated with lower anxious states and higher release–tension and playful states. This pattern may reflect differences in interaction style, such as calmer handling, greater sensitivity to subtle behavioural cues, or allowing dogs more space to regulate their behaviour, factors that may co-vary with professional maturity or experience. This finding contrasts with earlier work reporting no association between handler experience and canine cortisol or behaviour [26], suggesting that professional characteristics may influence affective expression even in the absence of detectable physiological changes.

Living arrangements also appeared relevant in AAT. Dogs living in foster families rather than with handlers showed lower enthusiastic, anxious, and playful states, alongside higher levels of release–tension states, indicating that frequent context switching and reduced routine stability may require greater behavioural regulation during work. This interpretation aligns with recommendations emphasising predictable routines to minimise stress in working dogs [97].

In AAC, handlers’ unfamiliarity with the dog was associated with higher tense states, underscoring the role of relational safety in coaching-oriented interactions. While the importance of the handler–dog relationship for performance and welfare is widely acknowledged [31,98,99], empirical evidence linking unfamiliar handlers to dogs’ affective experiences has been limited. The present findings, therefore, provide empirical support for professional guidelines emphasising the need for in-depth knowledge of each individual dog’s preferences, capacities, and limits [1,2].

### 4.8. Client-Related Associations with Affective State

Client characteristics were modestly associated with dogs’ affective states across AAS types, suggesting that human–dog interaction characteristics may vary with client age (Figure 2). Across several contexts, older client age was associated with lower levels of uncertainty or tension-related affective states, whereas younger client age was more often linked to uncertain, tense, or less playful states. These associations may reflect age-related interaction style, including predictability, intensity, and movement patterns. Previous research has shown that dogs may display more stress-related or displacement behaviours when interacting with younger children, particularly those under 12 years of age [100]. Similarly, Corsetti et al. [101] suggested that ambiguity or inconsistency in human–dog interactions may increase the likelihood of stress-related behaviours. Taken together, these findings support the interpretation that younger clients may—often unintentionally—create less-predictable interaction contexts, which could contribute to elevated uncertainty or tension in dogs. This highlights the importance of supervision, guidance, and age-appropriate structuring of interactions to support positive affective experiences for dogs in AASs.

### 4.9. Strengths and Limitations

A major strength of this study is its large and ecologically valid dataset. With 837 scored sessions involving 63 dogs and 30 handlers across four distinct AAS categories, it is one of the largest field datasets currently available in animal-assisted intervention research. This design reflects variations in workload, environment, and human–dog interaction with both professionals and clients, making the findings highly applicable to real-world practice.

Furthermore, to the best of our knowledge, this is the first study to apply Principal Component Analysis (PCA) to identify affective states in dog-assisted services. The PCA yielded coherent affective-state structures across all AAS categories (AAA, AAE, AAC, and AAT), accounting for 45–61% of the behavioural variance, with acceptable-to-good sampling adequacy (KMO = 0.59–0.73). This aligns with PCA applications in applied animal behaviour research (e.g., [51,52]).

Another methodological strength is the use of trained handlers as observers. All professionals received training and were tested before data collection, which improved both scoring consistency and behavioural awareness. This training component is itself relevant to animal welfare, as even experienced practitioners benefit from ongoing practice in recognising signals of positive and negative mental states. The use of structured ethograms and digital data collection further improved reliability and reduced data entry errors [102].

At the same time, retrospective behavioural scoring poses a limitation. Although it was chosen as the most feasible method for large-scale field research and practical implementation, consistent with approaches used in recent equine-assisted studies [53], it may be affected by recall bias. Behavioural observation also risks rater drift over time. While observers were trained prior to data collection, no repeated inter-rater reliability checks were conducted during the eight-week period, which may have affected longitudinal consistency. Future research should include repeated recalibration procedures to enhance reliability over time [103].

The composition of the professional sample should also be considered when interpreting the findings, as it was predominantly female and varied in experience. Individual characteristics, including gender, may influence how animal behaviour is perceived, for example, through differences in empathy or attentiveness to behaviour cues [81]. At the same time, previous research emphasises the importance of handler-related competencies, such as experience, training, and the ability to recognise and respond to stress signals, in accurately monitoring canine behaviour [31,104]. In the present study, all participating professionals were trained and met predefined competency criteria, which likely supported a consistent level of observational quality. Nevertheless, some variation in interpretation cannot be excluded, and caution is warranted when generalising the findings to more diverse professional populations.

Additionally, a notable limitation of this study is the absence of objective physiological data to complement the behavioural observations. While behaviours provide a rich source of information regarding a dog’s emotional experience, welfare is a multidimensional construct that is best captured through a combination of behavioural and physiological measures. The inclusion of biological markers, such as cortisol or heart rate variability, could have provided additional objective support for classifying the identified affective states and offered deeper insight into the underlying physiological arousal associated with different AAS contexts. Future research should aim to integrate these behavioural findings with physiological monitoring in field settings to further validate these affective components and enhance our understanding of the ‘win–win’ potential in animal-assisted services.

Overall, animal-assisted services can be ethically sound and mutually beneficial only when dogs’ individual characteristics, session design, environment, and professional qualities support fostering positive affective states, as emphasised in One Welfare and positive welfare frameworks [20,22,33].

## 5. Conclusions

This study suggests that dogs’ affective states in animal-assisted services (AAAs, AAE, AAC, and AAT) are shaped by a complex interplay of individual dog characteristics, session structure, and the social context in which the work takes place. By combining systematic behavioural observation with PCA-derived affective states and multiple regression analyses, this study offers an extensive, field-based perspective on how dogs may experience their work across different types of AAS.

Taken together, the results indicate that, within the observed sample, animal-assisted services do not appear to be inherently beneficial or inherently stressful for dogs. Instead, dogs’ emotional experiences appear to emerge from the interaction between individual predispositions, session design, and social relationships. This variability highlights the importance of tailored, context-sensitive approaches to AASs, in which dog selection, workload management, session structure, and professional continuity are carefully considered to support positive affective states and sustained engagement.

Finally, the field data generated in this study were also used to further validate expert-derived behavioural welfare thresholds for AAS contexts. Together, these complementary lines of research link observed affective states in real-world settings to practical welfare benchmarks, providing a preliminary foundation for more evidence-based monitoring, evaluation, and management of the well-being of dogs participating in AASs.

## Figures and Tables

**Table 1:** Table of behaviours assessed. Definitions and scoring categories of behaviours included in this study, with reference to the sources on which the definitions were based.

Behaviour Label	Definition	Scoring Categories	Sources
Wide, slow tail wag	A broad, slow, controlled movement of the tail. The tail swing, observed from the base of the tail, moves in the shape of a half circle (>90 degrees).	Absent: 0% of the session Occasionally: 1 to 25% of the session Frequently: more than 25% of the session	[55,56]
High posture	Breed-specific posture in which the following three behaviours are observed: elevated tail, elevated head, and ears oriented forward. These behaviours may vary in intensity but are clearly present.	Absent: 0% of the session Occasionally: 1 to 5% of the session Frequently: more than 5% of the session	[36]
Low posture	Breed-specific posture in which at least two of the following behaviours are observed: low tail, ears oriented backward, and/or flexed hind legs. At least two behaviours must be clearly visible.	Absent: 0% of the session Occasionally: 1 to 5% of the session Frequently: more than 5% of the session	[36]
Interaction with handler	The dog is in physical contact with the handler or looks at the handler while the distance between the dog’s nose and the handler’s head decreases. Alternatively, the dog follows or remains around the handler within a radius of two times the dog’s body length.	Absent: 0% of the session Occasionally: 1 to 25% of the session Frequently: more than 25% of the session	[45,57]
Interaction with client	The dog is in physical contact with the client or looks at the client while the distance between the dog’s nose and the client’s head decreases. Alternatively, the dog follows or remains around the client within a radius of two times the dog’s body length.	Absent: 0% of the session Occasionally: 1 to 25% of the session Frequently: more than 25% of the session	[36,45]
Out of sight	The dog is for an extended period (>3 min) in a location where the handler cannot observe the dog’s behaviour.	Absent: 0% of the session Occasionally: 1 to 25% of the session Frequently: more than 25% of the session	
Lying down	The dog is lying down and is not involved in the interaction or situation.	Absent: 0% of the session Occasionally: 1 to 25% of the session Frequently: more than 25% of the session	[27,58,59,60]
Panting	Increased respiration with an open mouth, where the tongue is visible inside or outside the mouth.	Absent: 0% of the session Occasionally: 1 to 5% of the session Frequently: more than 5% of the session	[36,37,61]
Avoidance/backing up	The dog moves its body or body part in the opposite direction of an offered stimulus (client, handler, object, or other stimulus).	Absent: 0 times throughout the session Occasionally: 1 to 2 times throughout the session Frequently: more than 2 times throughout the session	[27,41,52,62,63,64]
Yawning	A slow, deep inhalation visible through a widely opened mouth and jaws.	Absent: 0 times throughout the session Occasionally: 1 to 5 times throughout the session Frequently: more than 5 times throughout the session	[25,36,60,61,62,63,65]
Self-grooming	Self-care behaviour, including licking, scratching, and/or nibbling parts of the dog’s own body.	Absent: 0 times throughout the session Occasionally: 1 to 5 times throughout the session Frequently: more than 5 times throughout the session	[28,36,66]
High, stiff, fast tail wag	Repeated stiff (short-amplitude) and rapid side-to-side movement of the tail in a position higher than the neutral breed standard.	Absent: 0 times throughout the session Occasionally: 1 to 2 times throughout the session Frequently: more than 2 times throughout the session	[36,55,56]
Low, stiff, fast tail wag	Repeated stiff (short-amplitude) and rapid side-to-side movement of the tail in a position lower than the neutral breed standard.	Absent: 0 times throughout the session Occasionally: 1 to 2 times throughout the session Frequently: more than 2 times throughout the session	[55,56,67,68,69]
Sniffing the ground	The nose moves along the ground with clear sniffing movements, not related to food.	Absent: 0 times throughout the session Occasionally: 1 to 5 times throughout the session Frequently: more than 5 times throughout the session	[37,41,67,68,69,70]
Play behaviour	Behaviour related to play, such as play bows or play vocalizations, occurring individually (with or without a toy) or in interaction with a dog or human.	Absent: 0 times throughout the session Occasionally: 1 to 5 times throughout the session Frequently: more than 5 times throughout the session	[26,41,69,70,71,72,73,74]
Lip lick	A part of the tongue briefly becomes visible and moves sharply and directly along the lips and/or nose. Excluded when the dog is anticipating food or drink or has eaten or drunk within the previous minute.	Absent: 0 times throughout the session Occasionally: 1 to 10 times throughout the session Frequently: more than 10 times throughout the session	[36,37,52,61,75]
Body shake	Active shaking of the body involving more than just the head. Movements are more abrupt and larger than trembling.	Absent: 0 times throughout the session Occasionally: 1 to 5 times throughout the session Frequently: more than 5 times throughout the session	[25,36,67,76]
Slowed movement	Slowed body movement compared with the dog’s normal pace when moving from one point to another, possibly recognizable by incomplete movements.	Absent: 0 times throughout the session Occasionally: 1 to 2 times throughout the session Frequently: more than 2 times throughout the session	[77]
Vocalisation to person	Loud or subdued barking, growling, howling, whining, or squeaking while oriented toward a person. Not related to play, food, or a given command.	Absent: 0 times throughout the session Occasionally: 1 to 5 times throughout the session Frequently: more than 5 times throughout the session	[36,37,41,61,65,78]

**Table 2:** Component loadings of Principal Component Analysis (PCA) performed on the presence of the frequently observed behaviours during 255 sessions of animal-assisted activities (AAAs) with 22 dogs. The components are labelled according to the behaviours that contribute significantly to each component. For clarity, component loadings with absolute values ≤ 0.400 are omitted.

Behaviour/Label	Detached	Thoughtful	Uncertain	Playful
	Component 1	Component 2	Component 3	Component 4
Sniffing the ground	0.749			
Out of sight	0.733			
Lying down	0.619			
Self-grooming	0.540			
Lip lick	0.515		0.406	
Low, stiff, fast tail wag	0.421	0.497		
Interaction with client		0.705		
Wide, slow tail wag		0.618		
Low posture		0.549		
Panting		0.547		
Slowed movement		0.509		0.482
Body shake			0.640	
Interaction with handler			0.596	
High, stiff, fast tail wag			0.534	
Yawning			0.525	
High posture				0.718
Play behaviour				0.583
Avoidance/backing up				0.524
Variance explained	14.0%	11.8%	10.2%	9.1%
Eigenvalue	2.948	2.002	1.793	1.353

**Table 3:** Component loadings of Principal Component Analysis (PCA) performed on the presence of the frequently observed behaviours during 195 sessions of animal-assisted coaching (AAC) with 14 dogs. The components are labelled according to the behaviours that contribute significantly to each component. For clarity, component loadings with absolute values ≤ 0.400 are omitted.

Behaviour/Label	Playful	Comfortable	Tense	Anxious	Uncertain
	Component 1	Component 2	Component 3	Component 4	Component 5
Panting	0.872				
Low, stiff, fast tail wag	0.691				
Play behaviour	0.635				
Body shake	0.598				0.418
High, stiff, fast tail wag	0.593				
High posture	0.506		−0.504		
Yawning	0.448		0.504		
Wide, slow tail wag		0.810			
Lying down		0.746			
Self-grooming		0.486			
Lip lick			0.799		
Vocalisation to person			0.542		
Low posture				0.801	
Avoidance/backing up				0.785	
Sniffing the ground					−0.683
Interaction with handler					0.647
Variance explained	19.6%	11.7%	10.6%	9.7%	9.6%
Eigenvalue	3.531	2.273	1.583	1.321	1.098

**Table 4:** Component loadings of Principal Component Analysis (PCA) performed on the presence of the frequently observed behaviours during 71 sessions of animal-assisted education (AAE) with 7 dogs. The components are labelled according to the behaviours that contribute significantly to each component. For clarity, component loadings with absolute values ≤ 0.400 are omitted.

Behaviour/Label	Engaged	Uncertain	Anxious	Aroused
	Component 1	Component 2	Component 3	Component 4
Play behaviour	0.762			
Interaction with client	−0.614			
Wide slow wag	−0.591		−0.580	
Vocalisation to person	−0.585			
Lying down	0.551			
Sniffing the ground	−0.492	0.551		
Panting	0.430			
Low posture		0.703		
Low tight and fast wag		0.659		
Slowed movement		0.539		
Avoidance/backing up		0.503		
Interaction with handler			0.703	
Body shake			0.614	
Self-grooming			−0.585	
Lip lick			0.435	
High, tight and fast wag				0.855
High posture				0.775
Variance explained	17.0%	12.9%	12.0%	10.5%
Eigenvalue	3.232	2.317	1.838	1.508

**Table 5:** Component loadings of Principal Component Analysis (PCA) performed on the presence of the frequently observed behaviours during 316 sessions of animal-assisted therapy (AAT) with 38 dogs. The components are labelled according to the behaviours that contribute significantly to each component. For clarity, component loadings with absolute values ≤ 0.400 are omitted.

Behaviour/Label	Enthusiastic	Anxious	Release tension	Playful
	Component 1	Component 2	Component 3	Component 4
High, stiff, fast tail wag	0.780			
High posture	0.725			
Wide slow wag	0.650			
Interaction with client	0.597			
Body shake	0.532		0.501	
Self-grooming	−0.421		0.538	
Avoidance/backing up		0.713		
Lip lick		0.699		
Slowed movement		0.695		
Panting		0.511		
Low posture		0.434		
Yawning			0.597	
Lying down			0.577	
Vocalisation to person			−0.475	
Play behaviour				0.765
Interaction with handler				0.609
Variance explained	16.1%	13.7%	10.5%	9.4%
Eigenvalue	3.210	2.069	1.479	1.197

## Data Availability

The original contributions presented in this study are included in this article/Supplementary Materials. Further inquiries can be directed to the corresponding author.

## Supplementary Materials

The following supporting information can be downloaded at https://www.mdpi.com/article/10.3390/ani16071063/s1. Table S1: Descriptives of the sessions per type of intervention.; Table S2: Descriptives of the handlers for the type of intervention they are mostly active in.; Table S3: Descriptives of the dogs that worked per AAS category.; Table S4: Percentage of sessions in which each behaviour was displayed frequently, occasionally, or was absent during AAA sessions over a two-month period.; Table S5: Percentage of sessions in which each behaviour was displayed frequently, occasionally, or was absent in AAC over a two-month period.; Table S6: Percentage of sessions in which each behaviour was displayed frequently, occasionally, or was absent in AAE over a two-month period.; Table S7: Percentage of sessions in which each behaviour was displayed frequently, occasionally, or was absent in AAT over a two-month period.; Table S8: Scores on associations between affective states and explanatory variables for AAA.; Table S9: Scores on associations between affective states and explanatory variables for AAC.; Table S10: Scores on associations between affective states and explanatory variables for AAE.; Table S11: Scores on associations between affective states and explanatory variables for AAT.
