# Supplementary material for: Dogs’ Behavioural Responses to Dog-Assisted Interventions: A Field Study
Source: Animals (Basel). 2026 Mar 31;16(7):1063. doi: 10.3390/ani16071063 (PMC13072279; doi:10.3390/ani16071063)
Supplement: Supplementary file 1 [file animals-16-01063-s001.zip › animals-4179262-supplementary.pdf]

Supplementary material

Table S1. Descriptives of the sessions per type of intervention. The descriptives are given in percentage of sessions. AAA n = 255, AAC n = 195, AAE n = 71, AAT n = 316.

	AAA	AAC	AAE	AAT
Food is used	70%	70%	100%	83%
Materials are used	61%	77%	96%	60%
Dog on leash for the entire session	11%	6%	54%	9%
Dog on leash for most of the session	9%	12%	25%	8%
Dog on leash for less than half of the session	7%	19%	7%	13%
Session inside	49%	50%	75%	77%
Session outside	23%	37%	17%	14%
Session both in- and outside	5%	13%	8%	9%
Dog is familiar with surroundings	93%	98%	97%	94%
Client is unfamiliar with the dog	35%	8%	11%	22%

Table S2. Descriptives of the handlers for the type of intervention they are mostly active in. The descriptives are given in number of professionals or the average (standard deviation). n=30

	AAA	AAC	AAE	AAT
The professional is between 18 and 25 years old	1	1	0	1
The professional is between 26 and 30 years old	1	0	0	1
The professional is between 31 and 40 years old	3	1	0	3
The professional is between 41 and 50 years old	2	2	2	3
The professional is between 51 and 60 years old	1	1	1	4
The professional is 61 years or older	0	1	0	0
Average experience of the professional	2 (1,41)	3,5 (1,89)	3 (0,82)	5,08 (4,30)
The professional has an education in AAS	6	6	3	9
The professional has no education in AAS	2	0	0	2
The professional does not work with their own dog	1	1	0	0

Table S3. Descriptives of the dogs that worked per AAS category. The descriptives are given in number of dogs or the average (standard deviation). The total number of participating dogs was n=63, with several dogs participating in multiple AAS categories.

	AAA	AAC	AAE	AAT
Average age of the dog (STDV)	4,30 (2,70)	4,46 (2,87)	5,21 (2,97)	5,41 (2,48)
Average years of experience of the dog (STDV)	2,25 (1,35)	2,64 (1,88)	3,14 (1,81)	3,49 (2,30)
Male dog	7	5	2	18
Bitch	15	9	5	20
The dog started at less than 1 year of age	2	4	0	6
The dog started between 1 and 2 years of age	9	4	4	14
The dog started at more than 2 years of age	10	6	3	18
Companion and Toy Dogs	1	1	0	1
Sheepdogs and Cattledogs	7	3	2	4
Cross breeds	5	3	1	9
Scent hounds and related breeds	0	1	0	1
Pinscher and Schnauzer- Molossoid and Swiss Mountain and Cattledogs	0	1	1	2
Retrievers- Flushing Dogs- Water Dogs	4	4	2	18
Spitz and primitive types	1	0	0	1
Terriers	2	0	0	0
Pointing Dogs	2	1	1	2
Health deficiencies	3	2	2	5
No health deficiencies	18	12	5	32
The dog lives with a foster family	1	0	0	22
The dog lives with the handler	18	14	7	16

Table S4. Percentage of sessions in which each behaviour was displayed frequently, occasionally, or was absent during AAA sessions over a two-month period.

	AAA		
	absent	occasionally	frequently
Wide, slow tail wag	10%	62%	28%
High posture	61%	34%	4%
Low posture	85%	15%	0%
Interaction with handler	11%	59%	30%
Interaction with client	3%	45%	52%
Out of sight	83%	11%	6%
Lying down	65%	26%	9%
Avoidance/ backing up	68%	30%	2%
Yawning	79%	21%	1%
Self-grooming	79%	18%	2%
Panting	60%	25%	14%
High, stiff, fast tail wag	70%	18%	11%
Low, stiff, fast tail wag	86%	9%	4%
Sniffing the ground	48%	28%	23%
Play behaviour	57%	38%	4%
Lip lick	46%	51%	2%
Body shake	59%	40%	0%
Slowed movement	88%	9%	2%
Vocalisation to person	79%	18%	3%

Table S5. Percentage of sessions in which each behaviour was displayed frequently, occasionally, or was absent in AAC over a two-month period.

	AAC		
	absent	occasionally	frequently
Wide, slow tail wag	27%	46%	27%
High posture	79%	20%	1%
Low posture	92%	6%	1%
Interaction with handler	17%	78%	6%
Interaction with client	0%	23%	78%
Out of sight	93%	5%	1%
Lying down	34%	41%	24%
Avoidance/ backing up	77%	22%	2%
Yawning	68%	30%	2%
Self-grooming	60%	39%	2%
Panting	60%	29%	12%
High, stiff, fast tail wag	58%	38%	5%
Low, stiff, fast tail wag	52%	14%	34%
Sniffing the ground	29%	64%	7%
Play behaviour	41%	46%	14%
Lip lick	46%	53%	1%
Body shake	71%	29%	1%
Slowed movement	90%	11%	0%
Vocalisation to person	75%	26%	1%

Table S6. Percentage of sessions in which each behaviour was displayed frequently, occasionally, or was absent in AAE over a two-month period.

	AAE		
	absent	occasionally	frequently
Wide, slow tail wag	59%	23%	18%
High posture	55%	41%	3%
Low posture	93%	4%	0%
Interaction with handler	0%	85%	15%
Interaction with client	0%	66%	34%
Out of sight	99%	0%	0%
Lying down	15%	63%	20%
Avoidance/ backing up	86%	13%	0%
Yawning	69%	30%	0%
Self-grooming	45%	52%	3%
Panting	76%	20%	4%
High, stiff, fast tail wag	58%	39%	3%
Low, stiff, fast tail wag	97%	3%	0%
Sniffing the ground	54%	45%	1%
Play behaviour	18%	79%	1%
Lip lick	58%	42%	0%
Body shake	25%	73%	1%
Slowed movement	87%	13%	0%
Vocalisation to person	94%	6%	0%

Table S7. Percentage of sessions in which each behaviour was displayed frequently, occasionally, or was absent in AAT over a two-month period.

	AAT		
	absent	occasionally	frequently
Wide, slow tail wag	5%	69%	26%
High posture	71%	28%	1%
Low posture	81%	17%	2%
Interaction with handler	1%	85%	15%
Interaction with client	0%	43%	58%
Out of sight	97%	4%	1%
Lying down	23%	54%	25%
Avoidance/ backing up	67%	26%	7%
Yawning	53%	47%	1%
Self-grooming	70%	28%	2%
Panting	57%	37%	7%
High, stiff, fast tail wag	72%	10%	18%
Low, stiff, fast tail wag	82%	15%	3%
Sniffing the ground	29%	52%	18%
Play behaviour	65%	26%	9%
Lip lick	55%	45%	0%
Body shake	58%	42%	0%
Slowed movement	86%	14%	1%
Vocalisation to person	79%	18%	4%

Table S8. Scores on associations between affective states and explanatory variables for AAA

AAA	***Detached***	***Thoughtful***	***Uncertain***	***Playful***
Session-related variables				
Session_duration	*0.04 ****		*0.02 **	
No use of food	*0.20 ****	*0.09 **		
No use of materials		*-0.06 **		
Dog attended day care setting (ref= worked)	*0.08 **		*-0,03*	
Not worked before (ref= worked before)	*-0.05 ***		*0.06 ***	
Number_dogs			*-0.06 **	
Number_persons		*0.01 **		
Dog-related variables				
Dogs age	*0.01 ****			
Dogs gender_female		*-0.34 ****	*0.10 ****	*-0.11 ****
Years of experience of dog		*0.19 ****	*-0.06 ****	*0.08 ****
Starting age of dog				*-0.18 ****
No health conditions (ref= orthopedic condition)		*0.20 ****	*-0.24 ****	*0.22 ****
F1 (Fearfulness)	*0.00 ***	*-0.01 ****		
Sheep dogs and cattle dogs relevel (ref = "Cross")		*0.20 ****		
Retrievers, Flushing dogs and Water dogs (ref = "Cross")		*0.17 ***		
Spitz and primitive types (ref = "Cross")		*-0.93 ****		
Pointing dogs (ref= "Cross")		*0.23 **		
Client-related variables				
Clients age	*-0.01 ***		*-0.01 ****	*-0.01 ***
Client is unfamiliar with dog				*0.07 ***
(Intercept)	*0.24 ****	*0.55 ****	*0.91 ****	*0.61 ****
	(0.02)	*(0.06)*	*(0.05)*	*(0.06)*
N	*161*	*161*	*161*	*161*
R2	*0,7*	*0,55*	*0,42*	*0,38*
*** $p < 0.001$; ** $p < 0.01$; * $p < 0.05$.				

Table S9. Scores on associations between affective states and explanatory variables for AAC

AAC	*Playful*	*Comfortable*	*Tense*	*Anxious*	*Uncertain*
Session-related variabels					
Session_duration	*0.04 ****				
No use of food				*-0.09 ***	
No use of materials				*0.23 ****	
Dog_leashed	*-0.04 ****		*0.04 **	*0.03 **	
Dog is unfamiliar with environment					*-0,16*
Not worked before (ref= worked before)				*0.05 **	
Number_dogs		*-0.09 ***		*-0.08 ****	
Dog-related variables					
Dogs age	*-0.07 ****	*0.24 ****		*0.09 ****	*-0.11 ****
Years of experience of dog			*0.04 ****		
No health conditions (ref= orthopedic condition)		*-0.49 ****			
Companion and toy dogs (ref = "Cross")	*-0,15*	*0.86 ****		*0.35 **	*-0.87 ****
Sheep dogs and cattle dogs relevel (ref = "Cross")	*0.17 ****	*-0.46 ****		*-0.31 ***	*0.35 ****
Scent hounds and related breeds (ref = "Cross")	*-0.22 ****	*0.97 ****		*0.40 ****	*-0.49 ****
Pinschers, Schnauzers, Molossoids and Swiss mountain cattle dogs (ref = "Cross")	*0.36 ****	*-1.70 ****		*-0.55 ****	*0.59 ****
Retrievers, Flushing dogs and Water dogs (ref = "Cross")	*0.12 ****	*-0.12 ****		*0*	*0*
Handler-related variables					
Professional is unfamiliar with dog			*0.53 ****		
Client-related variables					
Clients age			*-0.04 ****		
Client is unfamiliar with dog	*0.05 ***	*0.07 **			
(Intercept)	*0.67 ****	*-0,12*	*0.57 ****	*-0,21*	*1.15 ****
	(0.08)	*(0.12)*	*(0.05)*	*(0.13)*	*(0.10)*
N	*70*	*70*	*70*	*70*	*70*
R2	*0,73*	*0,85*	*0,45*	*0,36*	*0,52*
**** p < 0.001; ** p < 0.01; * p < 0.05.*					

Table S10. Scores on associations between affective states and explanatory variables for AAE

AAE	**Engaged**	**Anxious**	**Uncertain**	**Aroused**
Session-related variabels				
Session_duration			-0.17 *	
No use of materials	-0.24 *		0.23 **	
Worked outside(ref=inside)	-0.29 *	-0.53 ***		0.31 **
Worked both inside as outside (ref= inside)	-0,07	-0.50 ***		0,28
Number_persons		0.01 *	0.02 **	0,01
Dog-related variables				
Dogs age	-0.54 ***	-0.68 ***	0.91 ***	
Dogs gender_female	-3.07 **	-4.67 ***	5.34 ***	0.51 ***
Sheep dogs and cattle dogs (ref = "Cross")	-1.06 ***	-1.38 ***	1.58 ***	
Pinschers, Schnauzers, Molossoids, and Swiss mountain cattle dogs (ref = "Cross")	0.61 ***	0,06	-0.72 ***	
Retrievers, Flushing dogs and Water dogs (ref = "Cross")	0,14	0.71 ***	-0.19 *	
Client-related variables				
Client is unfamiliar with dog			-0.13 *	
Age of client			-0.07 *	0.13 *
(Intercept)	6.04 ***	7.67 ***	-8.11 ***	-0.52 **
	(1.55)	(1.06)	(1.76)	(0.16)
N	59	59	59	59
R2	0,71	0,64	0,77	0,47
*** p < 0.001; ** p < 0.01; * p < 0.05.				

Table S11. Scores on associations between affective states and explanatory variables for AAT

AAT	**Enthousiastic**	**Anxious**	**Release tension**	**Playful**
Session-related variables				
Mean number of sessions per week	-0.07 ***			
No use of food				-0.13 ***
No use of materials	-0.07 **		0.08 **	
Dog_leashed			0.05 **	
Dog is unfamiliar with environment	-0.12 **		0.16 ***	-0.14 **
Worked outside (ref= inside)	0,01	-0.09 *		
Worked both inside as outside (ref= inside)	0.10 **	0,07		
Dog attended day care setting (ref= worked)			0.26 ***	
Temperature environment				-0.01 **
Number_dogs				0.03 *
Number_persons				0.03 **
Dog-related variables				
Dogs age	-0.03 **			
Years of experience of dog	0.04 **			0.02 *
Starting age of dog	0.13 ***	0.05 **		0.07 ***
F1	0.00 ***	0.01 ***		0.01 ***
F2				0.01 **
F3	0.00 ***		-0.00 ***	0.01 **
F4		-0.00 **		
F5		0.01 ***		
Handler-related variables				
Age of professional		-0.05 ***	0.03 ***	0.03 **
Education level University (master) (ref= bachelor university of applied sciences)		-0.34 ***		-0.38 ***
Living at foster family (ref= living with professional)	-0.25 ***	-0.11 **	0.16 ***	-0.15 ***
N	157	157	157	157
R2	0,58	0,5	0,49	0,6
(Intercept)	-0,08	0,24	0.48 ***	-0,43
	(0.12)	(0.14)	(0.08)	(0.29)
***** p < 0.001; ** p < 0.01; * p < 0.05.**				
